# Antimicrobial susceptibility in *Clostridioides difficile* varies according to European region and isolate source

**DOI:** 10.1093/jacamr/dlae112

**Published:** 2024-07-23

**Authors:** Jane Freeman, Virginie Viprey, Duncan Ewin, William Spittal, Emma Clark, Jon Vernon, Warren Fawley, Georgina Davis, Valerija Tkalec, Mark Wilcox, Maja Rupnik, Kerrie Davies, COMBACTE-CDI Consortium, Marc Bonten, Kerrie A Davies, Mark H Wilcox, Ed Kuijper, Maja Rupnik, Sebastian Wingen-Heimann, Evelina Tacconelli, Tuba Vilken, Nicola Petrosillo

**Affiliations:** Healthcare Associated Infections Research Group, Leeds Institute of Medical Research, University of Leeds, Leeds, UK; Department of Microbiology, Leeds Teaching Hospitals NHS Trust, Leeds, UK; European Society of Clinical Microbiology and Infectious Diseases (ESCMID), Study Group for Clostridioides difficile (ESGCD), Basel, Switzerland; Healthcare Associated Infections Research Group, Leeds Institute of Medical Research, University of Leeds, Leeds, UK; Healthcare Associated Infections Research Group, Leeds Institute of Medical Research, University of Leeds, Leeds, UK; Healthcare Associated Infections Research Group, Leeds Institute of Medical Research, University of Leeds, Leeds, UK; Healthcare Associated Infections Research Group, Leeds Institute of Medical Research, University of Leeds, Leeds, UK; Department of Microbiology, Leeds Teaching Hospitals NHS Trust, Leeds, UK; Healthcare Associated Infections Research Group, Leeds Institute of Medical Research, University of Leeds, Leeds, UK; Department of Microbiology, Leeds Teaching Hospitals NHS Trust, Leeds, UK; C. difficile Ribotyping Network for England and Wales, UK Health Security Agency, Leeds, UK; Healthcare Associated Infections Research Group, Leeds Institute of Medical Research, University of Leeds, Leeds, UK; National Laboratory for Health, Environment and Food (NLZOH), Centre for Medical Microbiology, Maribor, Slovenia; Faculty of Medicine, University of Maribor, Maribor, Slovenia; Healthcare Associated Infections Research Group, Leeds Institute of Medical Research, University of Leeds, Leeds, UK; Department of Microbiology, Leeds Teaching Hospitals NHS Trust, Leeds, UK; European Society of Clinical Microbiology and Infectious Diseases (ESCMID), Study Group for Clostridioides difficile (ESGCD), Basel, Switzerland; European Society of Clinical Microbiology and Infectious Diseases (ESCMID), Study Group for Clostridioides difficile (ESGCD), Basel, Switzerland; National Laboratory for Health, Environment and Food (NLZOH), Centre for Medical Microbiology, Maribor, Slovenia; Faculty of Medicine, University of Maribor, Maribor, Slovenia; Healthcare Associated Infections Research Group, Leeds Institute of Medical Research, University of Leeds, Leeds, UK; Department of Microbiology, Leeds Teaching Hospitals NHS Trust, Leeds, UK; European Society of Clinical Microbiology and Infectious Diseases (ESCMID), Study Group for Clostridioides difficile (ESGCD), Basel, Switzerland

## Abstract

**Objectives:**

*Clostridioides difficile* epidemiology is evolving with country-associated emerging and resistant ribotypes (RT). Antimicrobial susceptibility testing of *C. difficile* isolated from clinical and animal samples collected across Europe in 2018 was performed to provide antimicrobial resistance data and according to *C. difficile* RTs and source.

**Methods:**

Samples were cultured for *C. difficile* and isolates PCR ribotyped. Metronidazole, vancomycin, fidaxomicin, moxifloxacin, clindamycin, imipenem, tigecycline, linezolid, rifampicin and meropenem minimum inhibitory concentrations (MICs) for 280 clinical and 126 animal isolates were determined by Wilkins–Chalgren agar dilution.

**Results:**

Fidaxomicin was the most active antimicrobial (all isolates geometric mean MIC = 0.03 mg/L) with no evidence of reduced susceptibility. Metronidazole MICs were elevated among RT027 (1.87 mg/L) and RT181 clinical isolates (1.03 mg/L). RT027 and RT181 had elevated geometric mean moxifloxacin MICs (14.49 mg/L, 16.88 mg/L); clindamycin (7.5 mg/L, 9.1 mg/L) and rifampicin (0.6 mg/L, 21.5 mg/L). Five isolates (RT002, RT010 and RT016) were metronidazole resistant (MIC = 8 mg/L) and 10 (RT027; RT198) had intermediate resistance (4 mg/L). Metronidazole MICs were not elevated in animal isolates. Increased geometric mean vancomycin MICs were observed among RT078, mostly isolated from animals, but there was no resistance (MIC ≥ 4 mg/L). Clinical and animal isolates of multiple RTs showed resistance to moxifloxacin and clindamycin. No resistance to imipenem or meropenem was observed.

**Conclusion:**

Increased antimicrobial resistance was detected in eastern Europe and mostly associated with RT027 and related emerging RT181, while clinical isolates from northern and western Europe had the lowest general levels of resistance.

## Introduction

Antimicrobial resistance in *C. difficile* is a growing concern. As a colonizer of the gastro-intestinal tract of humans and animals, *C. difficile* may be exposed to selection pressures from multiple or sequential antimicrobial courses. Previous studies have identified multiple antimicrobial resistance markers in *C. difficile* PCR ribotypes (RTs), such as epidemic RT027 and highlighted emerging antimicrobial resistant RTs within specific geographical locations.^1^

There is increasing interest in animal and environmental sources of *C. difficile* and wider concerns about antimicrobial resistance within the food chain.^2–4^ The Combatting Bacterial Resistance in Europe—*Clostridioides difficile* infections (COMBACTE-CDI) project is an IMI2 Horizon2020 framework project that aimed to develop a detailed understanding of the epidemiology and clinical impact of CDI across Europe.^5^ This afforded an excellent opportunity to examine the epidemiology of human and animal derived strains that were collected as part of this study to provide detailed and recent antimicrobial resistance data.^6^ The present study describes the results of phenotypic antimicrobial susceptibility testing. The antimicrobial testing panel was selected to provide data that can be compared with previous baseline data on anti-*C. difficile* agents (vancomycin, metronidazole, fidaxomicin) and those with known resistance in *C. difficile* (clindamycin, moxifloxacin, imipenem, rifampicin), while including other relevant (linezolid, meropenem and tigecycline) antimicrobials.^1^

## Materials and methods

### Ethics

The COMBACTE-CDI study is registered under the ClinicalTrials.gov Identifier NCT03503474. Ethical approval was received from participating countries and from the University of Leeds for the overarching study (IRAS244784). The planning conduct and reporting of this study was in line with the 2013 Declaration of Helsinki. Informed consent was not required for the use of anonymized residual diagnostic material and data.^6^

### Material

The collection of human clinical samples was previously described.^6^ Briefly, all residual diarrhoeal faecal samples (from community and hospital patients) sent to 119 recruited testing facilities from 12 European countries, on two sampling days, during 2018 were collected, culture and PCR ribotyped.^6^ Contemporaneous *C. difficile* isolates were collected during the same period from animal faecal samples (mainly pigs and rodents) in similar European regions through the COMBACTE-CDI network practising veterinarians or authors of veterinary *C. difficile* publications. Participating countries represented European regions according to the United Nations Geoscheme as follows: the north: UK, Ireland and Sweden; the south: Greece, Italy and Spain; the east: Poland, Romania and Slovakia, and the west: Belgium (replaced by Austria for animal isolates), France and the Netherlands. Collected animal strains or samples were shipped to National Laboratory for Health, Environment and Food (NLZOH). *C. difficile* was isolated from animal faecal samples following enrichment for 5 days in brain heart infusion broth supplemented with 0.1% L-cysteine, 0.5% yeast extract, 0.1% taurocholic acid and *C. difficile* selective supplement (Oxoid, Basingstoke, UK), ethanol shock and cultured on selective medium (CHROMID *C. Difficile*, CHROMID^®^  *C. difficile*, bioMérieux, Marcy-l’Étoile, France). Before shipping animal *C. difficile* isolates from NLZOH to the University of Leeds for further testing, MALDI-TOF Biotyper System (Bruker, Bremen, Germany) was used to confirm *C. difficile* identification and crude agarose PCR ribotyping performed.^7^ All samples received at the University of Leeds were cultured for *C. difficile* on CCEY agar (E and O laboratories, Scotland) and isolates were subsequently typed by PCR ribotyping following the *C. difficile* Ribotyping Network of England and Northern Ireland protocol (CDRN, Leeds, UK).^8,9^

### Antimicrobial susceptibility testing

Metronidazole, vancomycin, fidaxomicin, moxifloxacin, clindamycin, imipenem, tigecycline, linezolid, meropenem and rifampicin minimum inhibitory concentrations (MICs) for 280 clinical and 126 animal isolates were determined by Wilkins–Chalgren agar dilution as previously described.^1^ Briefly, antimicrobial dilutions were prepared according to solvents and diluents listed in the CLSI guidelines.^10^  *C. difficile* isolates were cultured anaerobically at 37°C for 24 h in pre-reduced Schaedlers anaerobic broths before adjustment to McFarland 1.0 equivalence and multipoint inoculation onto antimicrobial-containing agar plates. Inoculated plates were incubated for 48 h at 37°C in an anaerobic environment before examination. MICs were defined as the lowest concentration (in mg/L) of an antimicrobial that inhibited the growth of *C. difficile*. Control strains were *C. difficile* NCTC 700057, *B. fragilis* NCTC and *C. difficile* E4 (PCR RT 010). Data are presented as the geometric means of MICs for each antimicrobial for all isolates, human clinical, animal isolates, per European regions and for the most common RTs (represented by at least five isolates). MICs for each antimicrobial and for each of the isolates are also available in Table S1 (available as Supplementary data at *JAC-AMR* Online).

MICs for each antimicrobial were scored as sensitive = 0, intermediate = 1 or resistant = 2 for each isolate according to published breakpoints used in the The *Clos*ER study, and added to generate a cumulative resistance score (CRS) for each isolate.^1,11,12^ Resistance breakpoints used for CRS calculation were as follows: MICs ≥ 8 mg/L for metronidazole, vancomycin, moxifloxacin and clindamycin (intermediate score if ≥4 mg/L); MICs ≥16 mg/L for imipenem and meropenem (intermediate score if ≥8 mg/L); MICs >4 mg/L for linezolid; MICs >1 mg/L for fidaxomicin; MICs >0.25 mg/L for tigecycline and MICs ≥16 mg/L for rifampicin (intermediate score if 0.008–16 mg/L). The average (and median) CRSs across all human or animal isolates and per European region are presented.

## Results

Two hundred and eighty clinical human isolates (81 distinct RTs) and 126 animal isolates (30 distinct RTs) were collected. RT distribution differed considerably between clinical and animal sources. RT181 was the most represented isolated clinical RT (9% of isolates), followed by RT027 (8%) and RT014 (6%). By contrast, among animal isolates RT078 accounted for over 32% of isolates, followed by RT126 (14%) and RT005 (8%). RT078 and highly related RT126 were the only RTs present in numbers >10 in both clinical and animal collections.

Fidaxomicin was the most active treatment agent (geometric mean for both clinical and animal isolates: geometric mean MIC = 0.03 mg/L, Table 1) with no evidence of reduced susceptibility among the isolates in this collection (Table S1).

**Table 1.** Geometric mean MICs for all antibiotics tested against human clinical (*n* = 280) and animal *C. difficile* isolates (*n* = 126)

	Geometric mean MICs (mg/L)									
Isolates	MET	VAN	FDX	MXF	IMI	CLINDA	TIGE	LZD	RIF	MERO
Human	0.29	0.52	0.03	2.89	2.75	7.84	0.04	1.76	0.007	2.95
Animal	0.18	0.71	0.02	2.01	1.78	6.35	0.04	2.15	0.001	2.88
All	0.25	0.58	0.03	2.58	2.40	7.34	0.04	1.87	0.004	2.92

MET, metronidazole; VAN, vancomycin; FDX, fidaxomicin; MXF, moxifloxacin; IMI, imipenem; CLINDA, clindamycin; TIGE, tigecycline; LZD, linezolid RIF, rifampicin; MERO, meropenem .

Geometric mean metronidazole MICs (human isolates) were 0.29 mg/L but were elevated among predominating epidemic RT027 (1.87 mg/L) and eastern European-associated (Romania) RT181 (1.03 mg/L) (Table 2). RT027 and RT181 also had elevated geometric mean moxifloxacin MICs (14.49 and 16.88 mg/L, respectively); clindamycin (7.46 and 9.14 mg/L, respectively) and rifampicin (0.56 and 21.47 mg/L). Five human isolates belonging to RT002, RT010 and RT016 were metronidazole resistant (MIC ≥8 mg/L), while another 10 human isolates from eastern Europe (Poland, RT027 and RT198) showed reduced metronidazole susceptibility (MIC >2, <8 mg/L) (Table S1). Elevated metronidazole MICs were not observed in any animal isolates, including those from eastern Europe.

**Table 2.** Geometric mean MICs (mg/L) for all antimicrobials tested against clinical *C. difficil*e isolates representing common RTs (*n* > 5)

	Geometric mean MICs (mg/L)									
RT	MET	VAN	FDX	MXF	IMI	CLINDA	TIGE	LZD	RIF	MERO
001	0.29	0.44	0.01	7.46	3.48	14.81	0.05	1.41	0.001	2.83
002	0.20	0.79	0.04	1.06	1.78	7.55	0.04	1.59	0.001	3.36
003	0.17	0.50	0.04	1.35	3.62	4.88	0.05	2.00	0.001	2.97
005	0.30	0.30	0.03	2.00	2.21	6.56	0.04	2.00	0.001	2.69
009	0.16	0.63	0.04	1.12	1.78	8.98	0.04	1.41	0.001	2.64
010	0.61	0.41	0.04	1.35	1.64	23.78	0.04	1.90	0.001	3.28
014	0.18	0.41	0.03	1.12	1.59	8.00	0.05	2.00	0.001	2.45
015	0.14	0.50	0.03	1.12	2.24	3.56	0.04	2.00	0.006	2.83
018	0.19	0.21	0.01	9.19	3.03	9.19	0.03	1.52	0.439	3.48
020	0.19	0.64	0.03	1.66	2.27	7.05	0.05	2.00	0.001	3.31
027	1.87	0.79	0.03	14.49	4.13	7.46	0.05	1.54	0.557	4.27
039	0.29	0.55	0.04	2.00	3.62	26.25	0.03	1.81	0.001	3.60
046	0.19	0.57	0.02	4.59	4.00	36.76	0.06	2.00	0.001	2.64
078	0.18	0.69	0.05	2.64	1.91	4.81	0.04	1.72	0.001	2.09
106	0.21	0.65	0.05	4.00	3.67	6.17	0.05	1.83	0.001	3.67
181	1.03	0.51	0.03	16.88	4.69	9.14	0.03	1.70	21.47	4.00

MET, metronidazole; VAN, vancomycin; FDX, fidaxomicin; MXF, moxifloxacin; IMI, imipenem; CLINDA, clindamycin; TIGE, tigecycline; LZD, linezolid RIF, rifampicin; MERO, meropenem.

There were some small differences in geometric mean MIC between clinical and animal isolates (Table 1). While no instances of vancomycin resistance were recorded among all tested isolates, geometric mean vancomycin MICs were marginally higher in animal versus clinical isolates (0.71 versus 0.52 mg/L, respectively). Isolates belonging to RT078 showed a slightly higher geometric mean vancomycin MICs in animal isolates compared to human clinical isolates (0.78 versus 0.69 mg/L, Tables 2 and 3). RT078 was more commonly isolated from animals than humans (41 versus 15, respectively), but there was no vancomycin resistance (MIC ≥ 4 mg/L, Table S1).

**Table 3.** Geometric mean MICs (mg/L) for all antimicrobials tested against animal *C. difficile* isolates from common RTs (*n* > 5)

	Geometric mean MICs (mg/L)									
RT	MET	VAN	FDX	MXF	IMI	CLINDA	TIGE	LZD	RIF	MERO
005	0.16	0.81	0.02	1.52	1.62	14.93	0.04	2.46	0.001	4.00
014	0.16	0.76	0.05	1.52	1.00	12.13	0.03	2.00	0.001	3.48
045	0.18	0.66	0.01	5.66	2.00	51.98	0.05	2.64	0.001	2.00
078	0.21	0.78	0.03	2.41	2.03	4.58	0.05	2.21	0.001	2.67
126	0.16	0.93	0.02	2.42	2.00	5.44	0.03	2.24	0.001	2.52
695	0.22	0.50	0.02	1.15	1.52	0.13	0.06	2.00	0.001	2.30

MET, metronidazole; VAN, vancomycin; FDX, fidaxomicin; MXF, moxifloxacin; IMI, imipenem; CLINDA, clindamycin; TIGE, tigecycline; LZD, linezolid RIF, rifampicin; MERO, meropenem MIC.

**Figure 1.**
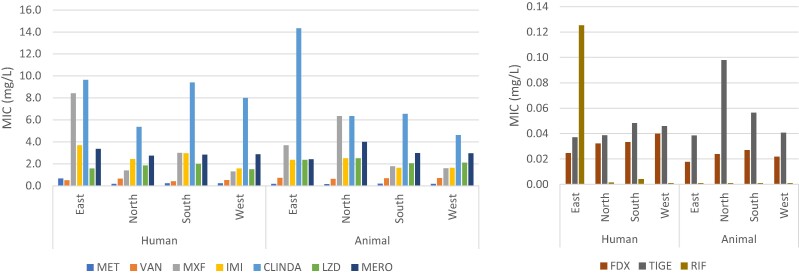
Geometric mean MICs for all antibiotics tested against human clinical and animal isolates by European regions. MET, metronidazole; VAN, vancomycin; MXF, moxifloxacin; IMI, imipenem; CLINDA, clindamycin; LZD, linezolid; MERO, meropenem; FDX, fidaxomicin; TIGE, tigecycline; RIF, rifampicin.

Moxifloxacin and clindamycin resistance (MIC ≥8 mg/L) was seen in both clinical and animal isolates (Figure 1a) and belonging to multiple RTs (Table 2 and Table 3). While geometric mean MICS were only marginally increased for clinical isolates over animal isolates (moxifloxacin = 2.89 versus 2.01 mg/L; clindamycin 7.84 versus 6.35 mg/L, Table 1), both clindamycin and moxifloxacin resistance were more evident among prevalent (*n* > 10) clinical RTs than prevalent animal RTs. Among prevalent clinical isolates, geometric mean moxifloxacin MICs were highest in RT181 (16.88 mg/L), RT027 (14.49 mg/L) (Table 2); while in prevalent animal isolates, only RT045 showed a modest increase in geometric mean MICs (5.66 mg/L, Table 3). Clindamycin resistance was seen across many clinically isolated prevalent RTs (RT181, RT014, RT010, RT039, RT001, RT009, RT018, RT046, Table 2) and in RT005, RT014 and RT045 in prevalent animal isolates (Table 3).

Geometric mean clindamycin MICs were highest in animal isolates in eastern Europe (Figure 1). Rifampicin MICs were elevated only in human clinical isolates, with resistance (MICs ≥16 mg/L) observed mainly among highly related RT181 and RT027 from eastern Europe (Figure 1, Table S1).

There was no evidence of imipenem or meropenem resistance (≥16 mg/L) in either clinical or animal isolates (Figure 1), however, geometric mean MICs were highest among clinically isolated highly related RT181 and RT027 from eastern Europe (imipenem = 4.72 and 4.36 mg/L; meropenem = 4.0 and 4.56 mg/L). Only three human isolates (RT001, RT027 and RT198) showed reduced susceptibility to tigecycline (>0.25 mg/L). Linezolid resistance (>4 mg/L) was observed in only four clinical isolates (RT010, RT012 and RT126) and two animal isolates (RT005 and RT126) (Table S1).

Only RT078 was prevalent (*n* > 10) in both clinical and animal isolate collections, however, closely related RT126 was present in both collections in smaller numbers (clinical = 4; animal = 18), along with RT014 (clinical = 18, animal = 5) and RT005 (clinical = 7, animal = 10). There was no evidence of resistance to any antibiotics tested for RT078 other than moxifloxacin (clinical isolates = 33% (5/15) versus animal isolates = 32% (13/41), MIC ≥8 mg/L) and clindamycin (clinical isolates = 53% (8/15) versus animal isolates = 36% (15/41), MIC ≥8 mg/L). Clindamycin resistance among animal isolates of RT078 were linked to eastern and southern Europe: geometric mean MICs were 5.44 and 6.35 mg/L, respectively, versus 2.75 and 2.0 mg/L for western and northern European isolates, respectively.

**Figure 2.**
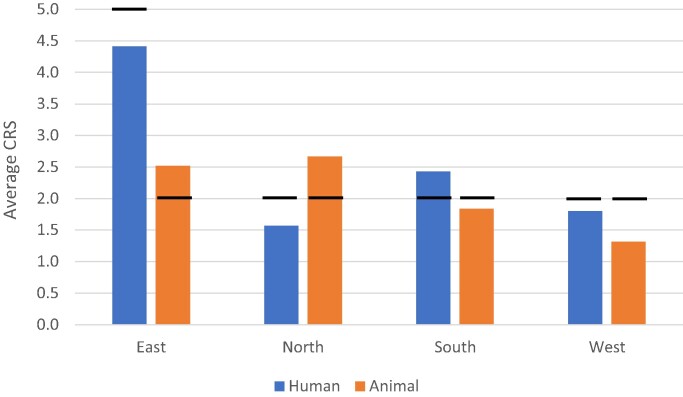
Antimicrobial resistance in clinical human and animal *C. difficile* isolates by European region. Average CRS is shown (median as black line).

Average (mean) and median CRS showed that resistance levels among clinical (but not animal) isolates were highest in eastern Europe (Figure 2).

## Discussion

There is increasing interest in the One Health approach to healthcare. The WHO particularly highlight the One Health approach as a potentially highly effective tool in combatting the spread of antimicrobial resistance.^13^  *C. difficile* is an organism that can be present in the gastro-intestinal tracts of humans and animals and in the environment, and as such is a prime example of the need for a One Health approach to antimicrobial resistance.^14^ The emergence of epidemic *C. difficile* RT027 in the early 2000s was notably associated with resistance to fluoroquinolones and, more recently, high-level resistance to cephalosporins has been also associated with this outbreak.^15,16^

The distribution of RTs among human clinical and animal isolates collected differed substantially in this study, with RT078 predominating in animals (>32% of isolates) and RT181 in human clinical isolates. The relatively high proportion of RT181 was influenced by the emergence of this RT in eastern Europe.^6^ Similarly high levels of emerging RTs (RT176 and RT198) were previously described in eastern European countries (Czech Republic and Hungary, respectively in a previous pan-European study of antimicrobial resistance).^1,17^ In both these cases, the emergent RTs also showed increased levels of antimicrobial resistance and were highly related to RT027, which was also prevalent in eastern Europe.^1^ RT176 has also been described as the predominant strain in Slovakia in 2018–2019.^18^ In the present study, most clinical isolates for RT027 (76%; 16/21) also came from eastern Europe. This indicates the continuing predominance of this RT in this region and the emergence of closely related RTs in particular locations within it. It is worth noting that even though isolates in our study were collected in 2018, these represent the most recent information at the European level as there is limited European-wide data on RT distribution and corresponding antimicrobial resistance testing available for more recent years. Indeed, the most recent CDI annual epidemiological report from the European Centre for Disease Prevention and Control for the period 2018–2020 describes RT data beyond 2018 from only two countries, in part due to lower surveillance reporting during the SARS-CoV-2 pandemic.^19^ Therefore, meaningful comparison of the data presented hereby with more recent years is not yet possible. Interestingly, two recent national studies conducted between 2016 and 2019 identified RT181 as the predominant strain in Greece and RT027 as the second most prevalent type in Denmark.^20,21^ This study presented the opportunity to examine and compare antimicrobial resistance of human and animal isolates from the same locations [albeit in smaller numbers (clinical versus animal isolates: east: 80 versus 25; north: 83 versus 3; south 82 versus 31; west 35 versus 67, average: 70 clinical versus 32 animal isolates)]. RT181 and RT027 isolates were, however, not identified in this animal collection, in accordance with previous studies.^22^ RT078 was the only RT present in both human clinical and animal collection in numbers >10 isolates, highlighting the association of this RT with both humans and animals.^2,3^

Resistance to antibiotics used in the treatment of CDI among both clinical and animal isolates was reassuringly low, with 15 clinical isolates demonstrating metronidazole MICs above the European Committee on Antimicrobial Susceptibility Testing breakpoint (>2 mg/L,^11^), but only five with MICs >4 mg/L, and no instances of vancomycin or fidaxomicin resistance. While geometric mean vancomycin MICs in animal derived RT078 isolates were slightly higher (0.78 mg/L), none displayed vancomycin resistance, which is uncommon among *C. difficile*.^1^

However, there was evidence of reduced susceptibility to metronidazole among RT027 and RT181 isolates from eastern Europe (GM MICs 1.87 and 1.03 mg/L, respectively). The mechanisms behind metronidazole resistance are only recently beginning to be understood, and the reasons for their emergence remain unclear.^23,24^ Decreased metronidazole susceptibility has been associated with clinical failures, but while it is no longer recommended as a first-line CDI treatment, it is still used.^25–27^ Surveillance of *C. difficile* RTs with this phenotype is therefore important, particularly in the light of poor gut metronidazole levels.^28^ Geometric MICs for clinical isolates were reflective of those observed in the large pan-European study by Freeman *et al*. and geometric mean MICs for animal isolates were similar.^1^ However, when general levels of resistance (to all antimicrobials) were assessed according to a CRS, these were higher in clinical isolates, and in particular those from eastern Europe. This is probably driven by RT027 and the emergent antibiotic resistant RT181 in Romania: together accounting for accounting for 51.3% (41/80) of human clinical isolates submitted from eastern Europe. While there were moxifloxacin and clindamycin resistant isolates in both clinical and animal collections, neither exhibited high-level resistance (>128 mg/L) such as that previously reported in epidemic human-associated RTs such as RT027 and 001,^1^ but high-level resistance to rifampicin was common among RT027 and RT181. Consumption rates of rifampicin and quinolones in Romania were among the highest submitted to ESAC-net in 2018.^29^ While this is in no way definitive evidence, it is possible that multiple antimicrobial selectors may contribute to the persistence and emergence of RTs in particular locations. There was much less evidence of multiple antimicrobial resistance among animal isolates; clindamycin resistance was mainly observed among RT005, RT014 and RT045 (GM mean MICs 14.93, 12.13 and 51.98 mg/L, respectively) and notably with RT045 animal isolates associated with eastern Europe (9/10). Interestingly, geometric mean clindamycin MICs were lower in clinical RT005 and RT014 isolates (6.56 and 8.00 mg/L, respectively). However, this is probably a reflection of origin: animal RT005 and RT014 were associated with western Europe (8/10 and 4/5, respectively), while clinical RT005 and RT014 were isolated in all four European regions. Only a single clinical RT045 was isolated, from eastern Europe, and this was clindamycin resistant. No antimicrobial resistance was detected in isolates of RT695, which was only identified in our animal collection from western Europe (five isolates from the Netherlands). Similarly, RT695 emerged as the most prevalent type in cattle from Dutch dairy farms in 2021.^30^

This study highlights the continued prevalence of antimicrobial resistant epidemic clinical *C. difficile* RT isolates, along with the emergence of closely related and similarly resistant RTs in particular geographic locations. While this may be linked to local antimicrobial prescribing policies, robust epidemiological and clinical studies are needed. Analysis of genome sequence data and continued surveillance will further enhance our knowledge of *C. difficile* resistance emergence and epidemiology.

## Supplementary Material

dlae112_Supplementary_Data

## Data Availability

The data presented in this study are available in this article and in Table S1.

## Transparency declarations

J.F. declares funding for research from Tillotts Pharma UK, Crestone, Pfizer and honoraria and funding to attend meetings from Tillotts Pharma UK. M.W. received consulting fees from Astra Zeneca, Debiopharm, Ferring, GSK, Nestlé, Paion, Pfizer, Phico Therapeutics, Qpex Biopharma, Seres, Summit, The European Tissue Symposium, Tillotts and Vedanta; lecture fees from GSK, Pfizer, Seres and Tillotts; and grant support from Debiopharm, GSK, Pfizer, Seres, Summit, The European Tissue Symposium and Tillotts. K.D. received research funding to the institute from Astellas Pharma Europe Ltd (EUCLID), bioMérieux, Pfizer, Techlab Inc., Sanofi Pasteur, Cepheid and Merck; and honoraria from Astellas Pharma Europe Ltd, Summit, Cepheid and Tillots. All authors report funding for the COMBACTE-CDI study as indicated in the funding statement.

## Supplementary data

Table S1 is available as Supplementary data at *JAC-AMR* Online.
